# Unidirectional Expiratory Valve Method to Assess Maximal Inspiratory Pressure in Individuals without Artificial Airway

**DOI:** 10.1371/journal.pone.0137825

**Published:** 2015-09-11

**Authors:** Samantha Torres Grams, Karen Yumi Mota Kimoto, Elen Moda de Oliveira Azevedo, Marina Lança, André Luis Pereira de Albuquerque, Christina May Moran de Brito, Wellington Pereira Yamaguti

**Affiliations:** 1 Department of Rehabilitation, Hospital Sírio-Libanês (HSL), São Paulo, São Paulo, Brazil; 2 Department of Pulmonary Function–Núcleo Avançado de Tórax (NAT), Hospital Sírio-Libanês (HSL), São Paulo, São Paulo, Brazil; The Hospital for Sick Children and The University of Toronto, CANADA

## Abstract

**Introduction:**

Maximal Inspiratory Pressure (MIP) is considered an effective method to estimate strength of inspiratory muscles, but still leads to false positive diagnosis. Although MIP assessment with unidirectional expiratory valve method has been used in patients undergoing mechanical ventilation, no previous studies investigated the application of this method in subjects without artificial airway.

**Objectives:**

This study aimed to compare the MIP values assessed by standard method (MIP_sta_) and by unidirectional expiratory valve method (MIP_uni_) in subjects with spontaneous breathing without artificial airway. MIP_uni_ reproducibility was also evaluated.

**Methods:**

This was a crossover design study, and 31 subjects performed MIP_sta_ and MIP_uni_ in a random order. MIP_sta_ measured MIP maintaining negative pressure for at least one second after forceful expiration. MIP_uni_ evaluated MIP using a unidirectional expiratory valve attached to a face mask and was conducted by two evaluators (A and B) at two moments (Tests 1 and 2) to determine interobserver and intraobserver reproducibility of MIP values. Intraclass correlation coefficient (ICC_[2,1]_) was used to determine intraobserver and interobserver reproducibility.

**Results:**

The mean values for MIP_uni_ were 14.3% higher (-117.3 ± 24.8 cmH_2_O) than the mean values for MIP_sta_ (-102.5 ± 23.9 cmH_2_O) (p<0.001). Interobserver reproducibility assessment showed very high correlation for Test 1 (ICC_[2,1]_ = 0.91), and high correlation for Test 2 (ICC_[2,1]_ = 0.88). The assessment of the intraobserver reproducibility showed high correlation for evaluator A (ICC_[2,1]_ = 0.86) and evaluator B (ICC_[2,1]_ = 0.77).

**Conclusions:**

MIP_uni_ presented higher values when compared with MIP_sta_ and proved to be reproducible in subjects with spontaneous breathing without artificial airway.

## Introduction

Maximal Inspiratory Pressure (MIP) is considered an effective method to estimate strength of inspiratory muscles [1,2]. This method has been widely used to evaluate the severity and follow-up of inspiratory muscle weakness in several clinical conditions [2–5], as well as for training load prescription and monitoring the outcomes of inspiratory muscle training programs [6–8]. In Intensive Care Units (ICU), MIP has also been used as a predictive index for successful weaning from mechanical ventilation [9,10] and, more recently, as a parameter for early detection of muscle weakness acquired in ICU [11].

The MIP evaluated by standard method (MIP_sta_) proposed by Black and Hyatt [1] is still the most common method for assessment of maximal respiratory pressures. In this method, MIP is quantified by maintaining the negative pressure for at least one second, against an occluded airway, after a forceful expiration near residual volume. Although this method has been considered well tolerated by patients and easy to perform, the measurement depends on the understanding and cooperation of individuals to perform really maximal respiratory efforts [2,12]. Low values (false positive diagnosis) are not uncommon and may represent poor technique of inspiratory effort instead of muscle weakness [13]. Furthermore, methodological variations such as number of necessary maneuvers, lung volume from which the maneuvers have been made, and types of equipment or interface may also compromise the reliability of measures [2,12], creating a discrepancy between the reference values [14–16].

To overcome the need for collaboration during MIP_sta_, Marini et al. [17] developed a method which shows optimization of inspiratory effort in critically ill and poorly cooperative patients undergoing mechanical ventilation. These authors proposed the use of a unidirectional expiratory valve, using low resistance to allow expiration in a selective way, while inspiration was prevented–MIP evaluated by unidirectional expiratory valve method (MIP_uni_). With inspiration blocked, respiratory efforts deflate the chest, making the patients start successive inspiratory efforts increasingly closer to residual volume, stimulating the generation of negative pressure. This method involves less patient-evaluator coordination because it represents a physiological response (increase of the respiratory drive after a prior insufficient inspiration), and can be used in patients unable to collaborate to perform the maneuver by MIP_sta_ [18].

Some authors [19,20] compared MIP_sta_ and MIP_uni_ in mechanically ventilated patients, and observed that MIP_uni_ was significantly higher when compared to MIP_sta_, demonstrating that MIP_uni_ optimizes inspiratory muscle capacity of action. However, to our knowledge, no study has reported using MIP_uni_ in subjects under spontaneous breathing without artificial airway. We hypothesized that the superiority of this method in the optimization of maximal inspiratory effort may also occur in these conditions, with a high reproducibility and better repeatability compared with MIP_sta_. In this context, this study aimed to compare MIP_sta_ and MIP_uni_ in subjects under spontaneous breathing without artificial airway. MIP_uni_ reproducibility and repeatability were also evaluated.

## Methods

### Subjects

We studied 31 subjects who met the inclusion criteria as follows: (1) age 18–60 years; (2) normal pulmonary function tests (FVC and FEV_1_ ≥ 80% of predicted and FEV_1_/FVC ≥ 0.7); (3) non-smokers; (4) absence of cardiopulmonary diseases; and (5) no prior contact with the methods tested. Exclusion criteria were: inability to carry out evaluations within the criteria for technical acceptability. The study was approved by the Sírio-Libanês Hospital Ethics Committee (HSL2011/17), and all subjects provided written informed consent.

### Set-up and measurements

Prior to MIP measurements, the subjects underwent assessment of personal history and lifestyle habits through a standard questionnaire, anthropometric evaluation, and pulmonary function test. The level of discomfort during the measurements in both MIP methods was also evaluated.

#### Pulmonary function test

The spirometry was performed using a portable digital spirometer (model Koko PFtesting; nSpire Healthy; Longmont; Colorado; USA), previously calibrated according to ATS and ERS recommendations [21]. The spirometric parameters were presented as absolute values and as a percentage of the predicted [22].

#### Maximal inspiratory pressure

MIP values were obtained with a digital vacuum manometer (model MVD500; Microhard; Porto Alegre; RS; Brazil). MIP_sta_ followed the Brazilian Society of Pulmonology and Phthisiology guidelines [12], using a digital vacuum manometer attached to a mouthpiece with a 2-mm diameter air-leak opening. MIP_sta_ was measured from the volume closest to residual volume by instructing the individuals to realize a forceful expiration followed by a maximal inspiration. For this evaluation, 10 maneuvers [23,24] were realized, respecting a rest period of one minute between them, in order to obtain three acceptable maneuvers including at least two repeatable ones. The highest value among the repeatable maneuvers was considered for the study.

**Fig 1:**
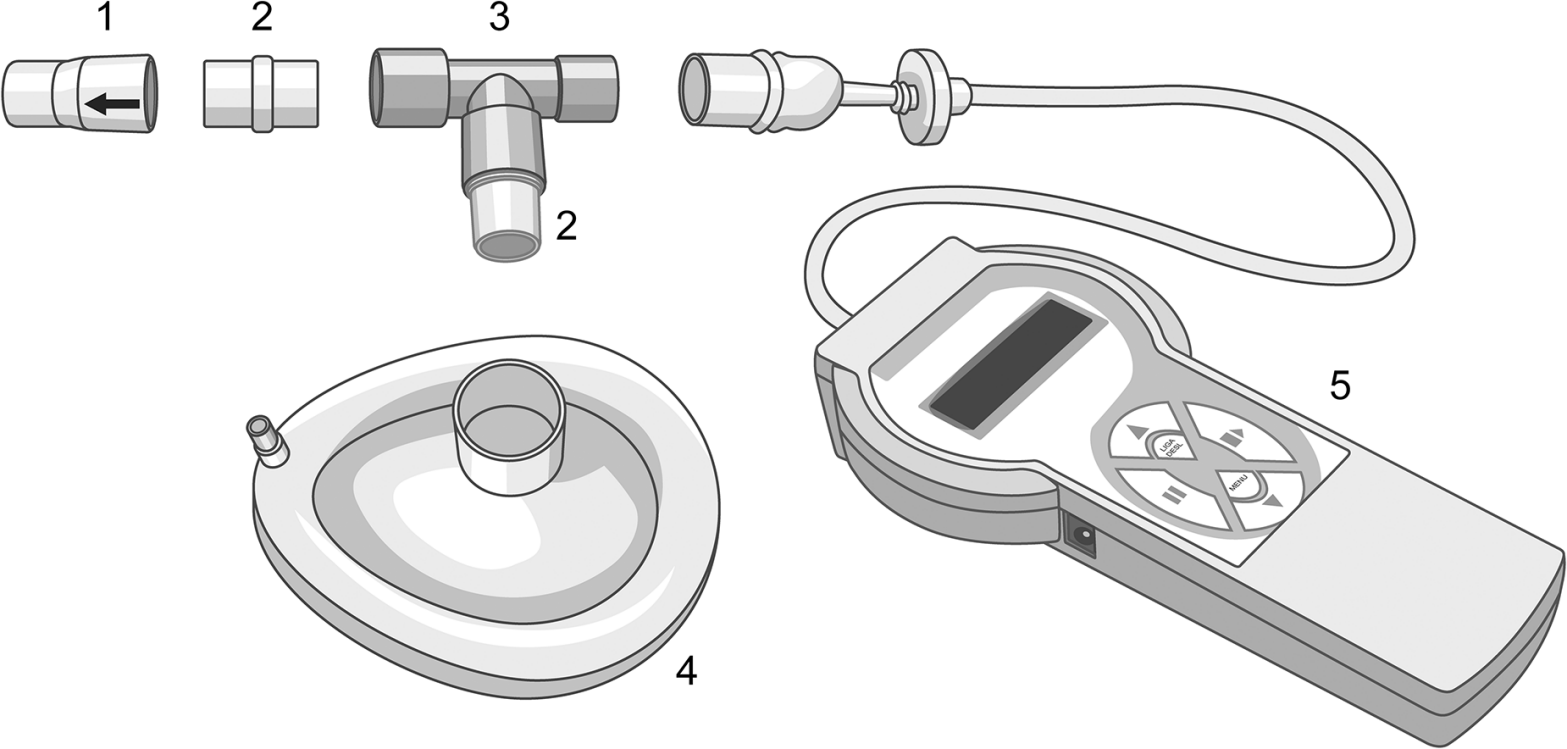
Materials used for MIP assessment by unidirectional expiratory valve method: (1) unidirectional expiratory valve; (2) straight connector; (3) T-tube; (4) face mask; (5) digital vacuum manometer.

MIP_uni_ was performed by using the digital vacuum manometer attached to a unidirectional expiratory valve and a face mask (Fig 1). The subjects were seated on a comfortable chair and remained attached to the mask for 20 seconds. During this period, all individuals were encouraged to make maximal respiratory efforts. For this evaluation, three maneuvers [19,20] were performed, respecting a rest period of one minute between them, and the highest value among the maneuvers was considered for the study.

#### Experimental protocol

**Fig 2:**
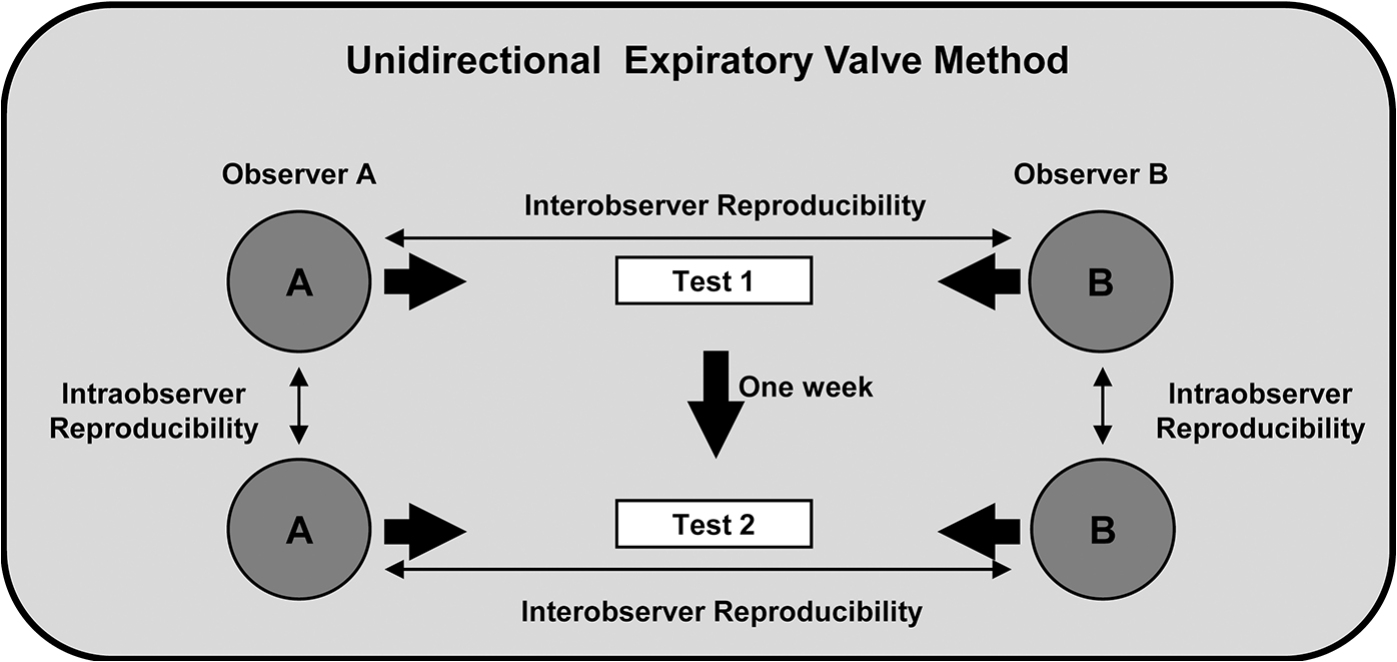
Process of MIP_uni_ assessment.

This study used a crossover design. MIP measurements were obtained by MIP_sta_ and MIP_uni_ in all subjects_,_ in a random order of application previously defined through a raffle. A 20-minute rest period was allowed between each method. MIP_sta_ was performed in a single moment (Test 1), and conducted by a single evaluator (evaluator A), who was kept blind to the results. In order to analyze the inter- and intraobserver reproducibility of MIP_uni_, this method was carried out by two evaluators (A and B), independently and in a random order, at two moments (Tests 1 and 2), at least one week apart (Fig 2). Repeatability was determined for each method (MIP_sta_ and MIP_uni_) considering the first and the last measurements from each participant obtained by evaluator A. The technical acceptability and recording of the values obtained in MIP maneuvers were performed by a third evaluator, so that evaluators A and B were kept blind to the results. The same conditions were maintained to perform MIP_uni_, both in Test 1 and Test 2: time of day, position, orientations and randomized order of the evaluators.

The discomfort caused during MIP assessment in both methods was measured by a visual analogue scale [25] of 10 cm, in which the "zero" point corresponded to "no discomfort", and point "ten" matched "maximum discomfort." The subjects were asked to mark a point on the scale, quantifying this subjective measure.

### Statistical analysis

Data were analyzed using SPSS for Windows, version 17.0 (IMB SPSS Statistics; IBM; Armonk; New York; USA). A sample size of 29 subjects was calculated using the results from a previous study [19] to detect a difference in MIP_uni_ of up to 14.06 with a standard deviation of 18.69 compared with MIP_sta_ (alpha value of 0.05 and a power of 0.8). Shapiro-Wilk test was used to analyze data distribution. The mean values of MIP_sta_ and MIP_uni_ were compared using the paired Student's t-test. This test was also used for comparing the mean values of MIP_uni_ for both evaluators (A and B), in both assessments (Tests 1 and 2). The inter- and intraobserver reproducibility of MIP_uni_ was established by the intraclass correlation coefficient (ICC_[2,1]_−a two-way random effects model with absolute agreement). The classification system by Munro [26] was used to interpret the ICC_[2,1]_: 0.0 to 0.25—little if any; 0.26 to 0.49—low; 0.50 to 0.69 –moderate; 0.70 to 0.89—high; 0.90 to 1.00—very high. Interobserver and intraobserver reproducibility was also evaluated by Bland-Altman plots [27] in order to better visualize the measurement agreement. To analyze repeatability of the values in both MIP_sta_ and MIP_uni_, the first and the last measurements of each participant were considered to calculate ICC_[2,1]_ in the first assessment (Test 1). The Bland-Altman repeatability coefficient [27] was also calculated for MIP_sta_ and MIP_uni_ values. The discomfort caused by both MIP_uni_ and MIP_sta_ was compared by using the Wilcoxon test. The significance level was established at 5%.

## Results

Thirty-one subjects were assessed for eligibility: 17 female and 14 male, with a mean age of 30.8 ± 6.2 years. Anthropometric characteristics and pulmonary function are shown in Table 1.

**Table 1:** Anthropometric characteristics and pulmonary function variables. n: number of subjects; kg: kilograms; m: meters; BMI: body mass index. FVC (% predicted): estimated percentage of predicted forced vital capacity; FEV_1_ (% predicted): estimated percentage of predicted forced expiratory volume in the first second; FEF 25–75% (% predicted): estimated percentage of predicted mean forced expiratory flow between 25% and 75% of FVC; VC (% predicted): estimated percentage of predicted vital capacity; IC: inspiratory capacity (L: liter); ERV: expiratory reserve volume.

Variables	Mean ± Standard deviation(n = 31)
Age (years)	30.8 ± 6.2
Body mass (kg)	71.8 ± 13.8
Height (m)	1.70 ± 0.08
BMI (kg/m^2^)	24.5 ± 3.5
Pulmonary function	
FVC (% predicted)	92.4 ± 12.1
FEV_1_ (% predicted)	93.3 ± 10.6
FEV_1_/FVC (% predicted)	101.0 ± 7.2
FEF 25–75% (% predicted)	95.1 ± 21.1
VC (% predicted)	90.1 ± 11.1
IC (L)	2.95 ± 0.63
ERV (L)	1.02 ± 0.45

### MIP_sta_ x MIP_uni_

**Fig 3:**
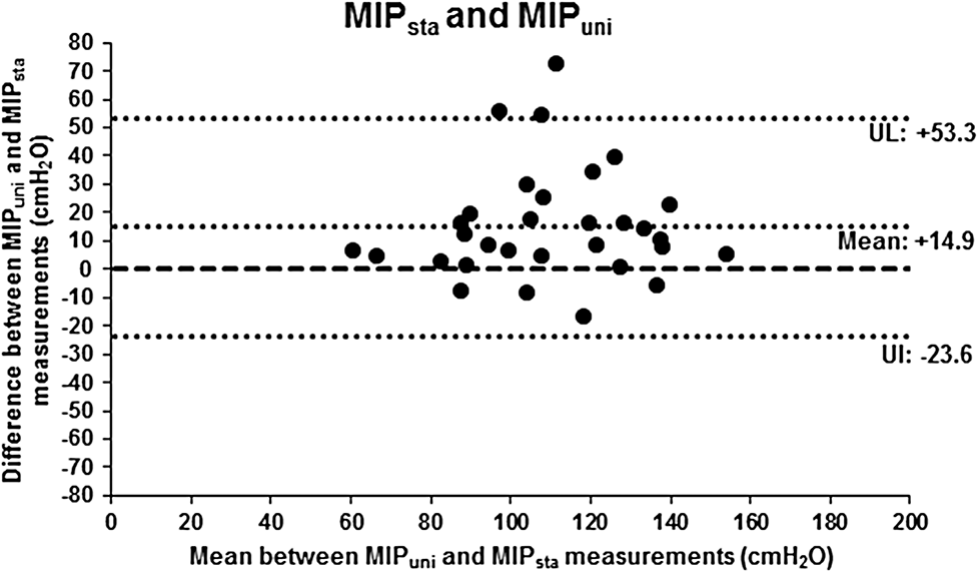
Bland-Altman plots of the agreement between MIP_sta_ and MIP_uni_ values (absolute values). MIP_sta_: Maximal inspiratory pressure evaluated by standard method; MIP_uni_: Maximal inspiratory pressure evaluated by unidirectional expiratory valve method; X axis: Mean of MIP values obtained by MIP_uni_ and MIP_sta_ for each subject of the study (MIP_uni_ value + MIP_sta_ value /2); Y axis: Difference between MIP values obtained by MIP_uni_ and MIP_sta_ for each subject (MIP_uni_ value–MIP_sta_ value); UL: Upper limit; LL: Lower limit.

MIP_sta_ (-102.5 ± 23.9 cmH_2_O) presented a statistically significant difference when compared to MIP_uni_ (-117.3 ± 24.8 cmH_2_O; p<0.001). MIP_uni_ was 14.9 ± 19.6 cmH_2_O above MIP_sta_ in absolute values (percentage difference mean of 16.9 ± 24.4%). By means of the Bland-Altman plots, a low agreement between MIP_sta_ and MIP_uni_ absolute values was observed, since the mean difference between obtained values was not close to zero. The dispersion of differences between values was also shown by Bland-Altman plots, with limits of agreement of -23.6 and +53.3 cmH_2_O (Fig 3). However, a significant positive linear correlation between the methods was observed: MIP_uni_ = (0.701 x MIP_sta_) + 45.53 (r = 0.68; p<0.001).

### Inter- and intraobserver reproducibility

MIP_uni_ values obtained by evaluators A and B in both tests are shown in Table 2. No statistically significant difference was found when comparing MIP_uni_ assessed by evaluators A and B, both in Test 1 (p = 0.19) and in Test 2 (p = 0.15). Also, no statistically significant difference was found when comparing MIP_uni_ ​​assessed by evaluator A, in Tests 1 and 2 (p = 0.10), and when comparing MIP_uni_ ​​assessed by evaluator B, in Tests 1 and 2 (p = 0.13).

**Table 2:** MIP evaluated by unidirectional expiratory valve method. n: number of subjects; cmH_2_0: centimeters of water.

		MIPMean ± Standard deviation
Test 1	Evaluator A	-117.3 ± 24.8
(n = 31)		
	Evaluator B	-114.9 ± 23.7
Test 2	Evaluator A	-113.5 ± 25.0
(n = 31)		
	Evaluator B	-110.2 ± 26.6

In the interobserver reproducibility evaluation, ICC_[2,1]_ was very high between MIP_uni_ obtained by evaluators A and B, in Test 1 (ICC_[2,1]_ = 0.91). In Test 2, ICC_[2,1]_ was high between the values obtained by these evaluators (ICC_[2,1]_ = 0.88). In the intraobserver reproducibility evaluation, ICC_[2,1]_ was high for both MIP_uni_ obtained by evaluator A (ICC_[2,1]_ = 0.86) and that obtained by evaluator B (ICC_[2,1]_ = 0.77) (Table 3).

**Table 3:** Inter- and intraobserver reproducibility of unidirectional expiratory valve method. ICC_[2,1]_: intraclass correlation coefficient; CI 95%: 95% confidence interval, *p*: level of significance.

		ICC_[2,1]_	CI 95%	*p*
Interobserver reproducibility	Test 1	0.91	0.83–0.96	<0.001
	Test 2	0.88	0.77–0.94	<0.001
Intraobserver reproducibility	Evaluator A	0.86	0.74–0.93	<0.001
	Evaluator B	0.77	0.57–0.88	<0.001

**Fig 4:**
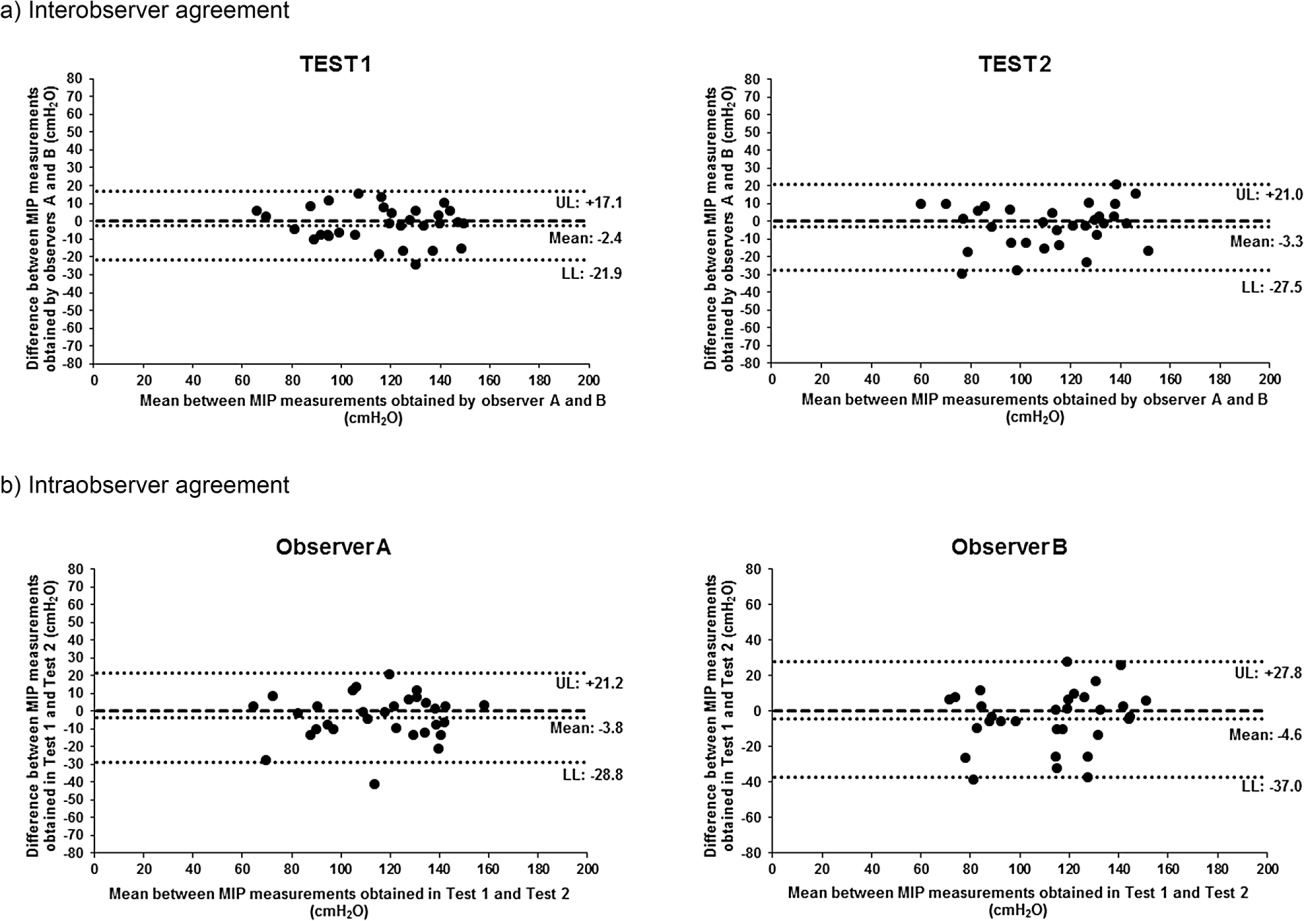
Bland-Altman plots of the inter- and intraobserver agreement between MIP_uni_ values (absolute values). a) X axis: Mean of MIP values obtained by evaluators A and B for each subject of the study (Value by evaluator A + Value by evaluator B /2); Y axis: Difference between MIP values obtained by evaluators A and B for each subject (Value by evaluator B–Value by evaluator A). b) X axis: Mean of MIP values obtained by evaluator A or B for each subject in Tests 1 and 2 (Value by evaluator A or B in Test 1 + Value by evaluator A or B in Test 2 /2); Y axis: Difference between MIP values obtained by evaluator A or B for each subject in Tests 1 and 2 (Value by evaluator A or B in Test 2—Value by evaluator A or B in Test 1). UL: Upper limit; LL: Lower limit.

The Bland-Altman plots showed the agreement between MIP_uni_ values obtained by evaluators A and B in both assessments (interobserver agreement), and also showed measurement agreement between MIP values obtained by each evaluator at two moments–Tests 1 and 2 (intraobserver agreement) (Fig 4).

### Repeatability of MIP_sta_ and MIP_uni_

MIP_sta_ repeatability assessment showed moderate ICC_[2,1]_ (ICC_[2,1]_ = 0.60). On the other hand, MIP_uni_ repeatability assessment showed very high ICC_[2,1]_ both by evaluator A (ICC_[2,1]_ = 0.94) and by evaluator B (ICC_[2,1]_ = 0.91) (Table 4).

**Table 4:** MIP measurement repeatability. ICC_[2,1]_: intraclass correlation coefficient; CI 95%: 95% confidence interval, *p*: level of significance.

		ICC_[2,1]_	CI 95%	*p*
	MIP_sta_	0.60	0.32–0.79	<0.001
Repeatability	MIP_uni_—Evaluator A	0.94	0.88–0.97	<0.001
	MIP_uni_—Evaluator B	0.91	0.83–0.96	<0.001

**Fig 5:**
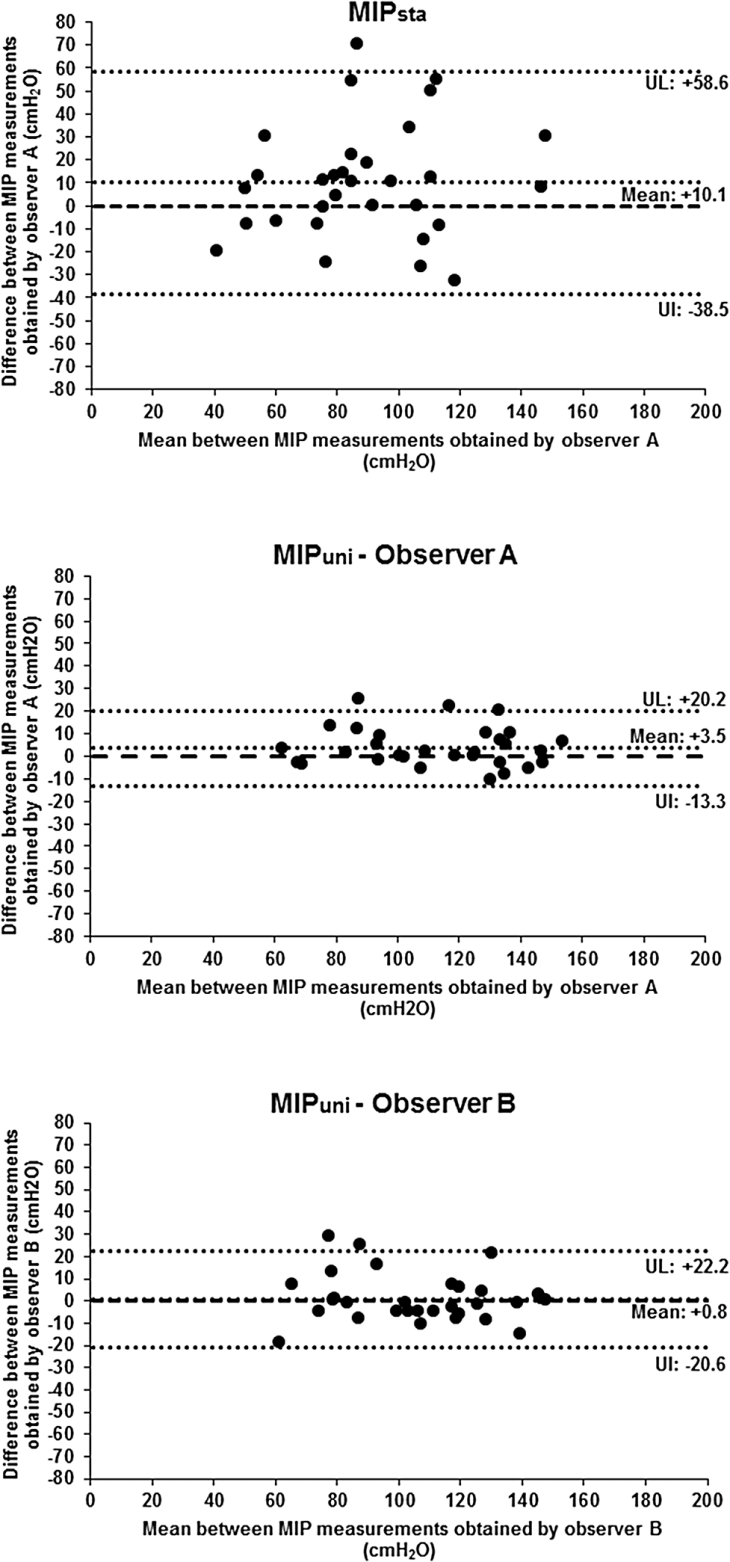
Bland-Altman plots of the agreement between MIP values obtained by standard method and by unidirectional expiratory valve method (absolute values). X axis: Mean between the first and last MIP values obtained by each method for each subject (First value + Last value /2); Y axis: Difference between the last and first MIP value obtained by each method for each subject (Last value–First value); UL: Upper limit; LL: Lower limit.

The Bland-Altman repeatability coefficient was 49.6 cmH_2_O for MIP_sta_. In MIP_uni_, the Bland-Altman repeatability coefficient obtained by evaluator A was 17.1 cmH_2_O and by evaluator B it was 21.8 cmH_2_O (Fig 5).

The discomfort reported during MIP_uni_ was higher (5.7 ± 2.8 cm) compared to MIP_sta_ (1.3 ± 1.6 cm; p<0.001).

## Discussion

The present study aimed to compare MIP_sta_ and MIP_uni_ in subjects under spontaneous breathing without artificial airway. The results showed significantly higher values of MIP_uni_ when compared to MIP_sta_ values in this population. Furthermore, MIP_uni_ proved to be an inter- and intraobserver reproducible method.

Previous studies [19,20] have also demonstrated superiority of MIP_uni_ when compared to MIP_sta_ but in patients with artificial airway. Possible explanations for the higher MIP values obtained by MIP_uni_ were mentioned by Caruso et al [19]. According to the authors, the respiratory drive increase during maneuvers might be due to the blockage of inspiration by using a unidirectional expiratory valve, which would cause carbon dioxide retention and subsequent release of chemical stimuli after the previous ineffective inspiration. In MIP_sta_, however, the respiratory drive would depend more on the collaboration of subjects than on physiologic response. Another plausible explanation is that, with the use of a unidirectional expiratory valve and the 20-second blockage of inspiration, patients could be forced to progressively reduce pulmonary volumes, performing inspiratory effort at a pulmonary volume closer to residual volume, optimizing the inspiratory muscle capacity of action (force–length relationship). It is important to mention that, both in MIP_sta and_ MIP_uni_, the negative pressure generated when the inspiratory effort is realized from the volume closest to residual volume reflects not only the pressure developed by the respiratory muscles, but also the passive elastic recoil pressure of the respiratory system including the lung and chest wall. According to the ATS [2], subjects find it easier to maximize their inspiratory efforts at low lung volumes; therefore, by convention and to standardize measurement, MIP is measured at or close to residual volume.

In the present study, it is worth noting that, for MIP_sta_ evaluation, a mouthpiece was used as interface, while in MIP_uni_ a face-mask was used. The superiority of MIP_uni_ cannot be attributed to the type of interface used, since previous studies have shown no significant difference between MIP values ​​obtained when using a mouthpiece or a face-mask [28,29].

With respect to the differences observed between MIP_sta_ and MIP_uni_, previous findings have shown that MIP_uni_ presented a variation of approximately 27–30% above MIP_sta_ [19,20]. In our study, the difference between the methods was 14.3%. This lower variation can be due to the differences in the population. The subjects of the present study were younger, healthy and without artificial airway. Older and hospitalized individuals, with artificial airway, such as those included in previous studies, could present less cooperation and worse performance during MIP_sta_ maneuvers.

We also aimed to assess the inter- and intraobserver reproducibility of MIP_uni_, showing that the method is reproducible. To determine the interobserver reproducibility of MIP_uni_, ICC_[2,1]_ showed high correlation between values obtained by different evaluators, when the same conditions were maintained during the assessment. Good agreement between values was observed using Bland-Altman plots, since the mean difference between values obtained by the evaluators was close to zero. Concerning the limits of agreement in the Bland-Altman plots, 95% of the difference between values obtained by both evaluators was less than 27.5 cmH_2_O. The study also assessed the intraobserver reproducibility and showed high ICC_[2,1]_ between values obtained by the same evaluator, at two moments, maintaining similar conditions. The dispersion of differences between values was also shown by Bland-Altman plots, with limits of agreement lower than 37 cmH_2_O. The same conditions were attempted in the present study for both Tests 1 and 2. Intraindividual variation factors, however, such as motivation during the test day, may have interfered with the results.

The repeatability of the values obtained by MIP_sta_ and MIP_uni_ was also compared. The repeatability analysis allows us to verify if the repeated measurements obtained by a single evaluator varied, when assessing the same subject with the same instrument, preserving identical conditions during a short period of time [26]. The ICC_[2,1]_ showed very high correlation between MIP_uni_ values while it showed moderate correlation between MIP_sta_ measurements. The repeatability coefficient for MIP_sta_ was 49.6 cmH_2_O, which means that 95% of the difference between the paired measures was up to 49.6 cmH_2_O. For MIP_uni_, the repeatability coefficient between measurements was lower, with less dispersion between the measurements (17.1 cmH_2_O for evaluator A and 21.8 cmH_2_O for evaluator B). The better repeatability of MIP_uni_ can be explained by a lesser coordination between subject and evaluator and a lesser learning effect [19].

### Limitations

A major limitation in our study was that MIP_sta_ was performed using a mouthpiece with a 2-mm diameter air-leak opening while in MIP_uni_ there was no air-leak opening in the face mask. Even though the use of an air-leak opening in the interface is recommended, this issue remains controversial in the literature. In a previous study, Smyth et al. [30] showed that the creation of a needle leak in the mouthpiece (18 gauge) had no effect on MIP for the prevention of glottis closure and artifactually high MIP. The authors suggest that careful instruction and observation of the subject may be more valuable than reliance on a small leak in the mouthpiece to prevent glottis closure. In the present study, a rigorous monitoring of the subjects was carried out in order to disregard maneuvers with evident signs of muscle contractions of the mouth and pharynx, rather than inspiratory muscles. In addition, our results showed a difference of more than 20 cmH_2_O for various individuals, even higher than 30 cmH_2_O in 6 subjects. It is very unlikely that such difference is due only to the absence of the air-leak opening. On the other hand, if the superiority of MIP_uni_ had been only due to the absence of the air-leak, we would not have observed individuals with lower MIP_uni_ values in relation to MIP_sta_, which is not true. We had around 6 individuals with lower MIP_uni_ values. Nevertheless, it is essential to conduct further studies aiming to verify the real influence of different sizes of orifices in determining MIP_uni_. Furthermore, the majority of subjects reported discomfort due to the interruption of inspiratory flow during MIP_uni_, which can be confirmed by higher values of discomfort evaluated quantitatively by the Discomfort Scale. However, only one subject reported mild headache after the test. Although no significant adverse effects were observed during maneuvers, future studies should be conducted to assess the feasibility and safety of this method in different clinical situations and comorbidities. In mechanically ventilated patients, Marini et al. [17] demonstrated that approximately 10 respiratory efforts or a 20-second rest period are needed after airway occlusion to obtain MIP_uni_. Further studies should investigate if a 20-second period is actually required to obtain MIP_uni_ in subjects under spontaneous breathing without artificial airway. A shorter time of attachment could minimize discomfort during this method.

## Conclusions

According to the present study results, it is evident that the evaluation by MIP_sta_ underestimates the inspiratory effort in patients without artificial airway. In this context, we recommend the use of MIP_uni_ to determine the strength of inspiratory muscles in individuals under spontaneous breathing without artificial airway, since this method presented higher MIP values, high inter- and intraobserver reproducibility and higher repeatability when compared to MIP_sta_. Considering that the normal reference values available [14–16] were determined by using MIP_sta_, further studies will have to establish new reference values of normality using MIP_uni_.
